# Impact of a stress coping strategy on perceived stress levels and performance during a simulated cardiopulmonary resuscitation: a randomized controlled trial

**DOI:** 10.1186/1471-227X-13-8

**Published:** 2013-04-22

**Authors:** Sabina Hunziker, Simona Pagani, Katrin Fasler, Franziska Tschan, Norbert K Semmer, Stephan Marsch

**Affiliations:** 1 Medical Intensive Care Unit, University Hospital Basel, University of Basel, Basel, Switzerland; 2 Department of Psychology, University of Neuchâtel, Neuchâtel, Switzerland; 3 Department of Psychology, University of Bern, Bern, Switzerland; 4 National Centre of Competence in Research on Affective Sciences, Geneva, Switzerland

**Keywords:** Stress, Cardiopulmonary resuscitation, Education, Simulation

## Abstract

**Background:**

Cardiopulmonary resuscitation (CPR) causes significant stress for the rescuers which may cause deficiencies in attention and increase distractibility. This may lead to misjudgements of priorities and delays in CPR performance, which may further increase mental stress (vicious cycle). This study assessed the impact of a task-focusing strategy on perceived stress levels and performance during a simulated CPR scenario.

**Methods:**

This prospective, randomized-controlled trial was conducted at the simulator-center of the University Hospital Basel, Switzerland. A total of 124 volunteer medical students were randomized to receive a 10 minute instruction to cope with stress by loudly posing two task-focusing questions (“what is the patient’s condition?”, “what immediate action is needed?”) when feeling overwhelmed by stress (intervention group) or a control group. The primary outcome was the perceived levels of stress and feeling overwhelmed (stress/overload); secondary outcomes were hands-on time, time to start CPR and number of leadership statements.

**Results:**

Participants in the intervention group reported significantly less stress/overload levels compared to the control group (mean difference: -0.6 (95% CI −1.3, -0.1), p=0.04). Higher stress/overload was associated with less hands-on time. Leadership statements did not differ between groups, but the number of leadership statements did relate to performance. Hands-on time was longer in the intervention- group, but the difference was not statistically significant (difference 5.5 (95% CI −3.1, 14.2), p=0.2); there were no differences in time to start CPR (difference −1.4 (95% CI −8.4, 5.7), p=0.71).

**Conclusions:**

A brief stress-coping strategy moderately decreased perceived stress without significantly affecting performance in a simulated CPR. Further studies should investigate more intense interventions for reducing stress.

**Trial registration:**

NCT01645566

## Background

Early start of effective cardiopulmonary resuscitation (CPR) significantly improves survival of patients following cardiopulmonary arrest in and outside the hospital [1,2]. While most studies in the past focused on technical aspects of CPR, recently the importance of non-technical factors such as teamwork, communication and leadership have been recognized [3-8]. Several studies have demonstrated that CPR causes significant mental stress in rescuers [9-15], and health care workers often feel unprepared to manage stress and conflicts in a cardiac arrest situation [16-18]. This is important because the stress experienced in an emergency situation may impair performance. In line with that, we recently found that feeling stressed and overwhelmed while delivering CPR was associated with worse CPR performance [14]. Hence, stress reducing measures may improve performance in critical situations.

To reduce stress, the focus on attentional processes may be a promising venue. Stress can have two opposite effects on attention. First, stress narrows attention [19]. For tasks that are relatively easy, narrowing attention can lead to improved performance by supporting a focus on the task [20]. However, narrowing of attention entails the danger of not noticing potentially important information, a phenomenon known as “tunnel vision” [21]. Furthermore, it is also related to premature closure, which is characterized by making decisions based on insufficient consideration of information available [22]. The second mechanism refers to an impaired ability to suppress irrelevant information, increased distractibility, ultimately leading to misjudgements of priorities [23-25]. A related mechanism refers to non-systematic scanning of informational cues [22]. However, information that distracts from task priorities may not only stem from external events but may also be generated internally, for instance by worrying about one’s own performance (intrusive thoughts) [26]. Such intrusive thoughts may be increased by noticing that one’s performance is not optimal [27], thus possibly leading to a vicious circle.

The response to acute stress is highly dependent on the individual’s perception of demands and resources [10,28], and on stress reactivity [29]. Therefore, stress management training may reduce stress [30]. Indeed, stress management training has had positive effects not only on stress indicators but also on performance [31,32]. However, although such procedures have been implemented in medical settings, they typically have not been evaluated in terms of medical performance [33].

Based on positive results achieved in other performance settings [15,32,34], and based on our recent finding that a brief leadership instruction improves CPR performance [5,35] we hypothesized that a brief task-focusing strategy may reduce stress and improve CPR performance. The aim of this study was therefore to (1) describe the stress patterns experienced during a CPR situation; (2) investigate whether the perceived stress was associated with CPR performance in terms of hands-on time and time to start CPR; (3) to investigate whether this task focusing strategy reduces perceived stress levels, and (4) whether this translates into better CPR performance. Based on findings that clear, directive leadership can enhance performance in cardiac resuscitation [5,8], we further (5) investigated if stress was associated with fewer leadership statements.

## Methods

### Participants and simulator

This study was conducted at the Simulator Center of the University Hospital in Basel, Switzerland, between December, 2007 and May, 2008. Workshops were offered to 4th year medical students and presented as a learning experience in a patient simulator. Prior to this simulation, no CPR training had been offered to the students within their medical curriculum. No information about the content of the scenarios and about our specific hypotheses was provided to students before the study (blinding).

The study was done in compliance with the Helsinki Declaration, approved by the local ethical committee (Ethikkommission beider Basel, EKBB, http://www.ekbb.ch/), and written informed consent was obtained from all participants.

For this study, we used a high fidelity manikin with the possibility of remote control of vital signs (Human Patient Simulator, METI) [36,37]. This full body simulator is a computer-based manikin with human physiology emulation capability that also can interact very realistically, e.g. by talking.

### Study design and intervention

**Figure 1:**
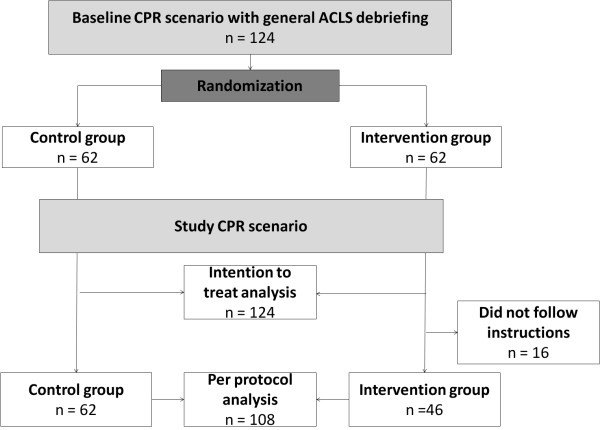
**Flow chart of randomisation groups.** N denotes number of participants.

This is a prospective randomized controlled study. Prior to the test-scenario, all students were made familiar with the simulator in a baseline training session followed by a general video-assisted debriefing focusing on ACLS algorithms (Figure 1). Students were then randomly allocated to two different randomization arms using computer generated randomization lists. Students in the control group did not receive any further instructions. Students in the intervention group received a 10 minute instruction to cope with stress. They were informed that an emergency situation is a stressful experience for health care workers and that perceived stress may interfere with their decision-making abilities and performance. Particularly, feeling overwhelmed by stress may cause cognitive impairment potentially leading to loss of concept how to deal with an emergency situation, which in turn further increases stress (vicious cycle). However, it is possible to overcome this situation by focusing on the basic conditions of the situation and the immediate actions that are needed. They were instructed that they should ask two task-focusing questions aloud (“what is the patient’s condition?”, “what immediate action is needed?”) to overcome the negative consequences of feeling overwhelmed by stress.

### Test-scenario

The test-scenario was a simulated witnessed cardiac arrest. Students performed the test-scenario alone. They were supported by a nurse, blinded to the experimental condition, who was instructed to be active on request only and not provide any information concerning CPR algorithms. When students entered the simulator room, the patient was conscious and responded to the questions of the students. Two minutes after the medical student started to take the medical history, the patient fainted and the monitor displayed ventricular tachycardia.

### Assessment of stress parameters

Upon completion of the simulation, perceived levels of stress and feeling overwhelmed were measured for different time points during the study period: (a) the baseline period immediately before resuscitation, (b) during the resuscitation period, (c) when the “patient” awakes, and d) during the debriefing period after the resuscitation. For each time point, we asked the students to quantify perceived levels of stress and feeling overwhelmed, measured on a Likert scale ranging from 1–20 (1 being lowest and 20 being highest). In a previous study, we found that perceived stress was best represented by a combination of these two items: feeling “stressed” and feeling “overwhelmed” [14]. We therefore combined the two items into a “stress/overload” index.

### Outcomes and measurements

The primary outcome was the average level of stress/overload during the resuscitation period for the experimental and the control group. Secondary outcomes were three performance measures, two relating to medical performance and one relating to team coordination. The two medical performance measures were: (a) hands-on time defined as duration of uninterrupted chest compressions and defibrillation in the first 120 seconds after the onset of the cardiac arrest. Each defibrillation was rated as 10 seconds of hands-on time. Interruptions of chest compressions to perform ventilation were rated as continuous hands-on time if the interruption was < 10 sec; (b) the time elapsed until CPR was started, defined as the time to the first meaningful measure (either defibrillation, chest compression or ventilation) after the onset of the cardiac arrest; the team coordination measure (c) was the number of leadership statements coded, using a predefined checklist containing the following categories based on previous research [5,7,8,38,39]: task assignment/task distribution, decision what to do, decision how to do, command. We also assessed the effectiveness of the instruction in the intervention group by investigating whether the two structuring questions were, indeed, asked aloud.

### Data analysis

Using frame-in-frame technology, the teams’ performance and the monitor displaying the “patient’s” vital signs were simultaneously recorded. Data to assess CPR performance measures and leadership statements were assessed based on the video-tapes recorded during simulation. More precisely, CPR-related actions were coded second by second; communication was transcribed, and each statement was coded as outlined above.

### Statistical analysis

Sample size calculations were based on assumptions from a previous observational study [14]. A study sample of 49 participants per randomisation arm gave the study a power of 90% to detect a relative 20% decrease in perceived stress/overload levels (from 12 to 10) assuming a two-tailed test, a 5% level of significance, and a standard deviation of ±3 in both groups. Assuming that 20% of participants would not follow the protocol, we included a total of 124 participants.

Discrete variables are expressed as counts (percentage) and continuous variables as means and standard deviation (SD). Students T-test were used for between condition comparisons. We also calculated linear regression models to investigate the association of the intervention with the primary and secondary outcomes. For all analyses we calculated an intention-to-treat analysis including all randomised students, and a per-protocol analysis considering only students that followed the instructions in the intervention group. All tests were two-tailed and P values < 0.05 were considered to indicate statistical significance. All analyses were performed using STATA 9.2 (Stata Corp, College Station, TX).

## Results

### Characteristics of participants

A total of 124 students (68% females) participated and were randomised to the intervention group (n=62) or to a control group (n=62). The groups were well balanced in terms of age and gender (Table 1). A total of 46 participants (74%) in the intervention group followed the instructions and posed the two questions aloud; these participants were included in the per-protocol analysis (Figure 1).

**Table 1:** Baseline characteristics of participants overall and within randomisation groups

	**All**	**Stress-intervention**	**Control group**	***P***
	**n=124**	**n=62**	**n=62**	
***Demographics***				
Age (mean, SD)	25.6 (2.1)	26.0 (2.5)	25.3 (1.6)	*0.1*
Female gender, n (%)	84 (68%)	42 (68%)	42 (68%)	*1.00*

### Stress/overload and performance

**Figure 2:**
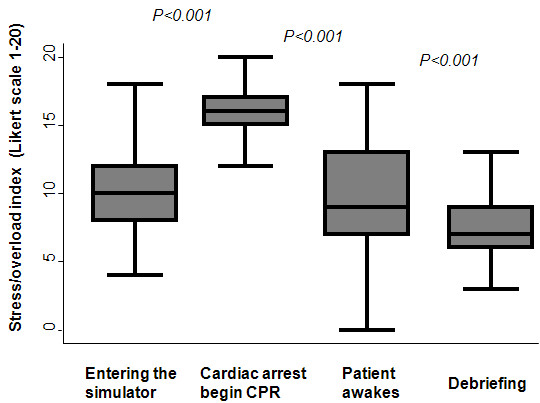
**Overall stress/overload at different time points during the CPR scenario.** Median lines are depicted; boxes represent the 25th to 75th percentile range and whiskers represent 5th and 95th percentiles.

Overall, the reported average mean stress/overload (scale 1–20) of participants was 10.9 (SD 1.8), and similar between male and female students (absolute difference -0.3 (95% CI −1.0, 0.4), p=0.80). Stress/overload levels significantly increased during the resuscitation period as compared to the two periods before and after resuscitation (Figure 2).

There was a significant negative correlation between the overall perceived stress/overload and hands-on time (r=−0.18, p<0.05) indicating that more stress/overload was associated with less hands-on time. No significant correlations were found between stress/overload and time to start CPR and number of leadership statements (data not shown).

### Impact of intervention on perceived stress

Overall, when considering all enrolled 124 students (Intention-to-treat analysis), participants in the intervention group reported significantly smaller amounts of perceived stress/overload compared to the control group (difference of mean perceived stress: -0.6 (95% CI −1.3, -0.1), p=0.04). When looking at stress levels at the different time points, no significant difference was found between the groups (see Table 2). The results were similar in a per-protocol analysis, where only the 46 participants who followed the instructions in the intervention group were included. Again, participants in the intervention group reported significantly lower mean amounts of perceived stress/overload overall, compared to the control group (difference −0.8 (95% CI −1.5, -0.1), p=0.02), but stress/overload levels were statistically different between the two groups only at the baseline time point before resuscitation.

**Table 2:** Association of intervention and overall stress and stress level at different time points

	**Intervention group**	**Control group**	**Difference (95% CI)**	***p***
**Stress/overload measures**	n=62	n=62		
*Intention-to-treat analysis (n=124)*				
**Overall stress/overload**				
Mean overall stress/overload during resuscitation, mean (SE)	10.6 (±0.21)	11.2 (±0.24)	−0.6 (95% CI −1.3, -0.1)	***0.04***
**Stress/overload during different time points**				
Stress/overload at baseline, mean (SE)	9.6 (±0.37)	10.6 (±0.42)	−1 (95% CI −2.1, -0.1)	*0.08*
Stress/overload during resuscitation, mean (SE)	15.9 (±0.34)	15.6 (±0.34)	0.2 (95% CI −0.7, -1.2)	*0.66*
Stress/overload when patients awakes, mean (SE)	9.3 (±0.41)	10.4 (±0.48)	−1.0 (95% CI −2.3, 0.2)	*0.1*
Stress/overload during debriefing, mean (SE)	7.4 (±0.36)	7.8 (±0.36)	−0.4 (95% CI −1.4, 0.6)	*0.42*
*Per-protocol analysis n=108*	n=46	n=62		
**Overall stress/overload**				
Mean overall stress/overload during resuscitation, mean (SE)	10.4 (±0.26)	11.2 (±0.24)	−0.8 (95% CI −1.5, -0.1)	***0.02***
**Stress/overload during different time points**				
Stress/overload at baseline, mean (SE)	9.1 (±0.37)	10.6 (±0.42)	−1.5 (95% CI −2.7, -0.4)	***0.01***
Stress/overload during resuscitation, mean (SE)	15.5 (±0.43)	15.6 (±0.34)	−0.1 (95% CI −1.2, 0.9)	*0.79*
Stress/overload when patients awakes, mean (SE)	9.4 (±0.52)	10.4 (±0.48)	−0.9 (95% CI −2.4, 0.5)	*0.18*
Stress/overload during debriefing, mean (SE)	7.5 (±0.42)	7.8 (±0.36)	−0.2 (95% CI −1.3, 0.8)	*0.66*

SE denotes standard error; 95% CI confidence interval, Intention-to-treat analysis includes all randomized participants, per-protocol analysis includes only participants who followed the instructions in the intervention group.

### Impact of intervention on performance

CPR was started after a mean of 43 sec (95% CI 39–46), and mean hands-on time in the first 120 sec overall was 55 sec (95% CI 51–59). On average, 11 leadership statements (95% CI 10–11) were recorded. There was a significant positive correlation between leadership statements and hands-on time (0.20, p=0.02) and a significant negative correlation between leadership statements and time to start CPR (r= -0.24, p<0.01). This indicates that participants with more leadership statements started earlier and did more uninterrupted CPR.

The intervention group had about 10% more hands-on time in the first 120 sec compared to the control group; this difference was, however, not statistically significant (57.8 sec (±3.28) vs 52.2 sec (±2.86), difference 5.5 (95% CI −3.1, 14.2), p=0.2). There were no differences between the two randomisation groups with regard to time to start CPR, particularly time to chest compression, ventilation and defibrillation (see Table 3). No differences between the groups also emerged for the number of leadership statements. The per-protocol analysis yielded similar results.

**Table 3:** Association of intervention and resuscitation performance

	**Intervention group**	**Control group**	**Difference (95% CI)**	***p***
**Performance measures**	n=62	n=62		
*Intention-to-treat analysis n=124*				
**Hands-on time (sec), mean (SE)**	57.8 (±3.28)	52.2 (±2.86)	5.5 (95% CI −3.1, 14.2)	*0.2*
**Time to first meaningful measure (sec), mean (SE)**	42 (±2.63)	43.3 (±2.43)	−1.4 (95% CI −8.4, 5.7)	*0.71*
Time to chest compressions (sec), mean (SE)	57.3 (±3.61)	60 (±3.26)	−2.8 (95% CI −12.4, 6.9)	*0.57*
Time to ventilation (sec), mean (SE)	67.6 (±3.68)	66.3 (±3.44)	1.4 (95% CI −8.6, 11.3)	*0.79*
Time to defibrillation (sec), mean (SE)	69.7 (±3.93)	66 (±3.84)	3.7 (95% CI −7.2, 14.6)	*0.54*
**Leadership statements, mean (SE)**	10.4 (±0.59)	10.9 (±0.56)	−0.5 (95% CI −2.1, 1.1)	*0.54*
*Per-protocol analysis n=108*	n=46	n=62		
**Hands-on time (sec), mean (SE)**	57.2 (±3.76)	52.2 (±2.86)	4.0 (95% CI −4.3, 14.1)	*0.29*
**Time to first meaningful measure (sec), mean (SE)**	42.5 (±3.07)	43.3 (±2.43)	−0.8 (95% CI −8.5, 6.8)	*0.82*
Time to chest compressions (sec), mean (SE)	59.2 (±4.22)	60 (±3.26)	−0.8 (95% CI −11.2, 9.6)	*0.88*
Time to ventilation (sec), mean (SE)	65.7 (±4.46)	66.3 (±3.44)	−0.5 (95% CI −11.5, 10.4)	*0.92*
Time to defibrillation (sec), mean (SE)	73.5 (±4.61)	66 (±3.84)	7.5 (95% CI −4.4, 19.3)	*0.21*
**Leadership statements, mean (SE)**	11.2 (±0.71)	10.9 (±0.56)	0.3 (95% CI −1.5, 2.1)	*0.72*

SE denotes standard error; 95% CI confidence interval, Intention-to-treat analysis includes all randomized participants, per-protocol analysis includes only participants who followed the instructions in the intervention group.

**Figure 3:**
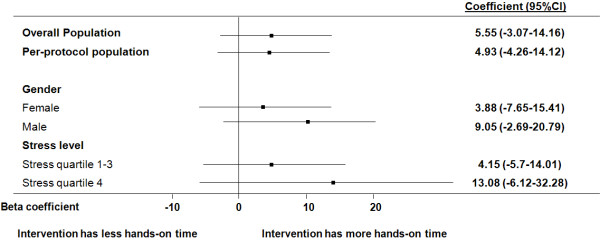
**Effect of intervention on hands-on time in different subgroups.** Coefficient relates to results of linear regression analysis including interaction terms for each subgroup. CI denotes confidence interval. Numbers refer to seconds of hands-on time within the first 120 seconds.

We also investigated the effect of the intervention in different subgroups (Figure 3). Male participants appeared to benefit more from the intervention compared to females (beta coefficient (95% CI) 9.05 (−2.69, 20.79) vs. 3.88 (−7.65, 15.41). Also, participants in the highest stress quartile appeared to benefit more from the intervention compared to participants in the lower quartiles (beta coefficient (95% CI) 13.08 (−6.12, 32.28) vs 4.15 (−5.7, 14.01). The effect of the intervention did not reach statistical significance in any of these subgroups.

## Discussion

This study investigated the influence of a short task-focusing strategy on perceived stress levels and performance of rescuers in a simulated CPR scenario. We found an increase in stress/overload levels during the resuscitation period and an association of stress/overload with CPR performance. Students in the intervention group reported less perceived stress/overload during the resuscitation period, and they had about 10% more hands-on time; performance differences did not, however, reach statistical significance.

Previous research has demonstrated that mental stress impairs performance of rescuers in emergency situations [14], which was also validated within this analysis. This may be due to several causal pathways. First, mental stress has been shown to impair the attentional resources because the cognitive system is in danger of becoming overloaded. During stressful situations, participants may selectively focus their attention to selected tasks only, thereby neglecting other potentially relevant information. As stress increases, the ability to filter out irrelevant information may decrease, leading to increased distractibility [23-25]. Second, studies found that stress impairs retrieval from memory; for example stress due to public speaking has been associated with impairments on tasks that required remembering previously learned information [40]; in our case, retrieval of existing knowledge about the treatment algorithm may have been impaired. Third, stress has also been shown to impair rational decision-making [10,25]. Finally, stress has also been implicated in loss of team perspective and decreased team performance [31,41]. Importantly, decreased performance due to stress may in turn further increase mental stress of rescuers leading to a vicious cycle.

Only few studies have evaluated the effectiveness of interventions to reduce chronic stress in medical practice. Effects of such interventions have included a reduction in perceived stress-levels for treatment groups [42,43], increased assertiveness scores [44,45], and increases in job satisfaction [46]. One study of behavioral training in general practitioners demonstrated a benefit in developing skills at coping with stress [30]. This training improved the general practitioner’s quality of work life and reduced their work-related psychological distress; yet, these were chronic stress situations and CPR related stress is an acute stress reaction. Similar stress coping strategies for acute emergency situations, such as CPR, are largely lacking. A recent German study investigated the effects of crew resource management (CRM) training including psychological teaching on the performance of intensive care professionals in a randomized-controlled trial [11]. The training did reduce stress, but no significant difference in the stress response or medical performance was noted in comparison to a group receiving traditional training; note that the CRM training was not specifically focusing on stress. Our intervention aimed at bringing the attention of rescuers to the important elements of the task and to task priorities by posing two task-focusing questions in case they felt overwhelmed by stress in a CPR situation. This intervention had a small but significant benefit in terms of perceived levels of feeling stress/overload; yet, no statistically significant improvement in performance was observed, although the intervention group did have 10% more hands-on time.

Although there is no definite explanation for the lack of association of our intervention with better performance, several explanations may be considered. First, this may be due to the relatively small sample size and lack of power. Although the intervention demonstrated a 10% relative increase in performance, this difference did not reach statistical significance; this was true overall and in different subgroups (Figure 3). Second, the intervention was very short, including only two questions the participants were expected to ask themselves. It is possible that the intensity of this intervention was not high enough to affect performance. This explanation is supported by the finding that stress levels, although decreasing overall during the resuscitation, did not significantly decrease during the most vulnerable and most stressful period, that is, when CPR was started; this was true in the intention-to-treat and the per protocol analysis. Thus, the intervention may not have been intense enough to influence stress levels to such a degree that stress-induced impairments of performance were successfully countered. Still, it has to be noted that the effect of the intervention on hands-on time was close to statistical significance (P = .059) in quartile of students that was most highly stressed. Furthermore, if the difference of 5.5 seconds in hands-on time between experimental and control group (and of 13.1 seconds in the most highly stressed quartile) can be confirmed in future studies, this would indicate a notable improvement in performance considering the low intensity of the intervention.

Interestingly, within this study we found that more leadership statements (such as commands, decisions what and how to do, task distribution among others) were associated with earlier start and longer duration of uninterrupted CPR performance. This validates previous observational research [8] and a randomized controlled trial that demonstrated a benefit from a brief leadership debriefing in terms of CPR performance [5,35]. Within the present trial, the task-focusing strategy did not increase the number of leadership statements, which may partly explain the lack of improvement in CPR performance. Perhaps a combination of stress-related and leadership-related instructions would yield stronger results.

This study has a number of limitations. The small number of participants included in this study limited the power of our analyses and increased the risk for type II errors. Although previous studies showed that participants rated the simulated resuscitation in a high fidelity simulator as highly realistic [36,37] and also perceived substantial stress [39], participants might still have perceived the simulated resuscitation as less stressful than a real life resuscitation. Participants were medical students and the results may not unconditionally be applied to more experienced intensive care physicians. Students were performing alone. Finally, only participants in the intervention group were made aware about the importance of stress during CPR and thus may have responded differently to the stress questionnaires (Hawthorne effect). Thus, measurement of performance is the preferred outcome measure and should be used in the future for similar research. As an alternative design, both groups could be made aware of stress but only one could receive a stress reduction intervention.

## Conclusions

A brief stress-coping strategy moderately decreased perceived stress without, however, significantly affecting performance of rescuers in a simulated CPR scenario strongly enough to yield a statistically significant difference. Further studies into the effect of stress and stress reducing strategies are warranted; they should consider an intervention that is still short yet somewhat stronger, for instance, by including not only questions but also self-guiding statements [47] and possibly a combination with instructions regarding leadership [5,35].

## Abbreviations

CPR: Cardiopulmonary resuscitation; ACLS: Advanced cardiac life support.

## Competing interest

All authors have no conflict of interests to disclose.

## Authors’ contributions

All authors have made substantial contributions to all of the following: (1) the conception and design of the study, or acquisition of data, or analysis and interpretation of data, (2) drafting the article or revising it critically for important intellectual content, (3) final approval of the version to be submitted. Particularly, Study concept and study design: SH, FT, NKS, SM; Data collection and data analysis: SH, SP, KF and SM; Drafting the initial version of the manuscript: SH, SP, and KF. All authors contributed to and approved the final version of the manuscript.

## Pre-publication history

The pre-publication history for this paper can be accessed here:

http://www.biomedcentral.com/1471-227X/13/8/prepub
